# Primary hyperaldosteronism: challenges in subtype classification

**DOI:** 10.1186/1756-0500-5-602

**Published:** 2012-10-30

**Authors:** Brian T Layden, Allison J Hahr, Dina M Elaraj

**Affiliations:** 1 Divisions of Endocrinology, Metabolism, and Molecular Medicine, Department of Medicine, Northwestern University Feinberg School of Medicine, Chicago, IL, USA; 2 Section of Endocrine Surgery, Department of Surgery, Northwestern University Feinberg School of Medicine, Chicago, IL, USA

**Keywords:** Hyperaldosteronism, Hypertension, Adrenal venous sampling

## Abstract

**Background:**

Primary hyperaldosteronism (PA) is a serious and potentially debilitating disease. Detailed guidelines have been written to guide endocrinologists in establishing the diagnosis of PA as well as in subtype classification of PA. The objective of this case report is to present a case where subtype classification of PA was challenging and repeated imaging of the adrenal glands helped establish the diagnosis in a patient with initial normal adrenal glands on CT and MRI images.

**Case presentation:**

We report a case of a 29-year-old woman with an established diagnosis of PA, but unclear subtype, who presented to us for further management. She initially presented for medical evaluation of uncontrolled hypertension and spontaneous hypokalemia 4 years prior. In the investigation of secondary causes of hypertension, plasma aldosterone-to-plasma renin activity ratio was elevated on two separate occasions, and primary hyperaldosteronism was confirmed by saline infusion test. Also during this time, she had adrenal venous sampling done 3 times at multiple institutions yielding confusing results. Initially, imaging by CT and MRI showed normal adrenal glands. To help establish the subtype of PA, we reimaged this patient’s adrenal glands one year later revealing a 2 cm left adrenal adenoma. Laparoscopic left adrenalectomy improved her hypertension and was curative of her hypokalemia.

**Conclusion:**

This case presents an unusual case where reimaging of the adrenal glands led to the discovery of a single adenoma, initially not observed on imaging studies.

## Background

Primary hyperaldosteronism (PA) is due to excess secretion of aldosterone by the adrenal glands. This hypersecretion (or uncontrolled secretion) in the majority of cases is from either aldosterone-producing adenoma (APA, or Conn’s syndrome) or bilateral adrenal hyperplasia [1]. Rarely, a patient may have ectopic aldosterone production, adrenocortical carcinoma, or glucocorticoid remediable aldosteronism. Because management of PA depends on the particular subtype of PA, distinguishing between these different types of PA is important [2]. Moreover, in the case of aldosterone-producing adenoma, surgical treatment is sometimes curative of hypertension and often curative of hypokalemia, an outcome that can be dramatic and allow the patient to avoid a lifetime of multiple medications and minimize the risk of complications from lifelong hypertension [3]. Here, we discuss an interesting case of PA where the radiographic studies were initially negative, but follow-up imaging 1 year later established the diagnosis of a single aldosterone-producing adenoma, which led to surgical cure for this patient.

## Case presentation

The patient is a 29 year-old woman who initially presented to her physician at the age of 25 with uncontrolled hypertension and hypokalemia. To control her blood pressure, multiple medications were required, with only minimal effect in establishing blood pressure control. Additionally, she had recurrent low potassium levels (less than 2.5 mEq/L) requiring large amounts of oral potassium chloride per day (120 MEq/day). Family history was significant for a deceased mother who had kidney failure at a young age from uncontrolled high blood pressure. Because of these above findings, she was sent to an endocrinologist for workup of secondary causes of hypertension.

The initial labs indicated possible PA by measuring plasma aldosterone concentration (PAC), plasma renin activity (PRA), and calculating an aldosterone-to-renin ratio (ARR) (PAC = 8.0 ng/dL, PRA = 0.1 ng/ml/h, ARR = 80 measured at 10 pm with potassium of 2.6 mEq/L). She was on multiple medications (labetalol and verapamil) at the time including spironolactone. Repeat labs indicated PAC = 16.0, PRA = 0.06, ARR = 267 measured at 10 am with potassium of 3.0 mEq/L, these labs were done after 6 weeks off of spironolactone. Of note, we had her take extra potassium tablets to obtain near-normal potassium levels before repeating these studies. Confirmation of PA was done by 2 liter saline infusion test over 4 hrs, where pre-infusion values showed a PAC/PRA ratio of 28/0.3 and post-infusion values showed a PAC/PRA ratio of 14/1.2 (potassium was 3.2 mEq/L at time of test, patient was off spironolactone for over 2 months prior to this test). Evaluation for other secondary causes of hypertension ruled out pheochromocytoma, Cushing’s syndrome, and renal artery stenosis.

During this period, she had dedicated adrenal imaging by both CT (3/23/2010) and MRI (7/13/2010) scan. These studies demonstrated normal adrenal glands, without evidence of nodules or hyperplasia (done 2 years after initial diagnosis, at age 27 years old). Adrenal venous sampling (AVS) was then performed to determine if lateralization of the aldosterone secretion to one adrenal gland occurred. However, the first AVS sampling was technically not successful. This procedure was subsequently repeated over the coming years twice more, and both times the serum potassium levels prior to the procedures were normal. While both procedures were technically successful, the results were difficult to interpret. These studies are highlighted in Table 1.

**Table 1:** Findings for the adrenal venous sampling procedures, where the right and left adrenal vein (AV) and inferior vena cava (IVC) values for aldosterone and cortisol are shown

**Year**	**Right AV aldosterone/cortisol**	**Left AV aldosterone/cortisol**	**Infrarenal IVC aldosterone/cortisol**	**Left-to-right lateralization ratio**
2010	127/200.8 = 0.63	338/242 = 1.4	70/21.4 = 3.3	2.2
2011	190/214 = 0.9	500/252.4 = 2.0	83/25 = 3.3	2.2

These values are obtained under continuous cosyntropin infusion. Prior to the procedure, her potassium levels were 3.7 mEq/L (for 2010 procedure) and 4.3 mEq/L (for the 2011 procedure).

**Figure 1:**
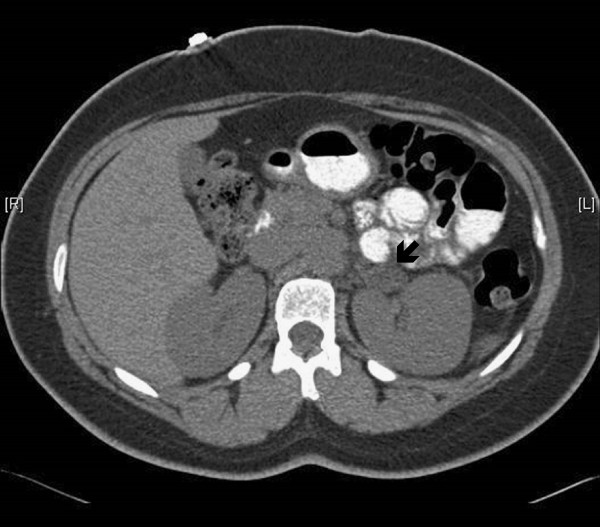
Adrenal CT scan of patient show left adrenal adenoma (see arrow).

Through the subsequent few years, the patient had multiple admissions to emergency rooms. Her presenting symptoms for these visits were mostly related to severe headaches, a symptom that developed when her blood pressure was uncontrolled. Some of these emergency room visits led to hospital admissions for further management of her uncontrolled blood pressure and/or hypokalemia. On one of the most recent admissions, we reimaged her adrenal glands (10/17/2011) and found a left adrenal adenoma (2.3 × 1.4 cm, less than 10 Hounsfield units on non-contrast CT images), a finding not previously observed approximately 1 year ago by CT scan and MRI (Figure 1). Considering her history, the authors discussed with her the pros and cons of left adrenalectomy and informed her that this is the likely source of excess aldosterone, though it could not be guaranteed. Due to her poor quality of life, which included multiple medications, intolerance to high dose potassium supplements, recurrent headaches and multiple hospital admissions, she decided to go forth with surgery. Pathology revealed adrenal cellular proliferation consistent with adrenal cortical adenoma. Post-operatively, she did well, and her blood pressure returned to normal limits on two anti-hypertensives. Aldosterone levels on post-operative day one were undetectable. She has maintained a normal blood pressure in the subsequent year on these two medications, including normal potassium levels off potassium supplementation on repeat blood studies. It has been 9 months since the operation, and at last visit, her blood pressure medications were decreased and one of these two medications will likely be able to be stopped altogether.

## Discussion

Primary hyperaldosteronism is thought to be a relatively common cause of hypertension, possibly accounting for 10% of cases [2]. An aldosterone-producing adenoma is an important cause of PA to identify, as surgery would be curative of PA and result in improvement or cure of hypertension and hypokalemia [2,4]. However, there are often difficulties in establishing not only the diagnosis of PA but also the subtype of PA.

Distinguishing unilateral from bilateral aldosterone hypersecretion is done by adrenal venous sampling, a challenging procedure that requires a level of expertise that is not always available [5,6]. In this particular case, AVS gave confusing results, because the cortisol levels should have been much higher in the adrenal veins than was detected, and the aldosterone/cortisol ratio is normally higher in the adrenal vein responsible for the PA than in the periphery. Although, the cortisol-corrected aldosterone ratios in each case showed some level of left lateralization, these data were insufficient to recommend left adrenalectomy. Moreover, these data highlight one of the major challenges in making this diagnosis, since not only is AVS a technically challenging procedure that is operator dependent, but interpretation of the results depends on appropriate specimen dilution by the laboratory [7]. Because adrenal macronodules are uncommon in young patients [8], and because her 2.3 cm left adrenal mass was new compared with imaging done 1 year prior, she proceeded with laparoscopic left adrenalectomy.

Other forms of primary hyperaldosteronism should always be considered, in particular glucocorticoid-remediable aldosteronism (GRA). In this case, the patient did have a family history of uncontrolled hypertension in her mother. However, further workup for GRA for this patient was not done because she had severe recurrent hypokalemia (less than 2.5 mEq/L), which is typically not seen in GRA [9]. Moreover, GRA usually presents at a very early age, often in childhood [9]. In addition to GRA, other inherited forms exist, including a recently described family with mutations in the *KCNJ5* gene, which leads to massive bilateral adrenal hyperplasia and severe hyperaldosteronism [10]. Of importance, somatic mutations of this gene may be an important factor in the development of aldosterone-producing adrenal adenomas [11].

As evident from her initial biochemical studies, this patient clearly had PA. However, initial imaging was negative for adrenal nodules. While small adenomas are not always visible on imaging studies [12], AVS often helps localize these lesions. However, three AVS procedures were performed and no precisely defined lateralization was established despite technically successful studies defined as an adrenal vein-to-periphery cortisol ratio of >5:1 [6]. A lateralization ratio of >4 is commonly used to define a unilateral source of aldosterone overproduction [5] and this patient’s lateralization ratio was 2.2. Cases of aldosterone producing adenomas with lateralization ratios <4 have been previously reported [13].

This case raises an important issue for clinicians that our clinical judgment should not be trumped by misleading results (as in this case with data from the AVS procedures and the early imaging studies). While we suspected that this patient had primary hyperaldosteronism based on her symptoms, the data were often hard to interpret. In this case, our persistence ultimately allowed us to make the diagnosis of a unilateral aldosterone-producing adenoma. It is important to follow guidelines, but in certain cases, clinical judgment should not be undervalued.

## Conclusion

Because aldosterone-producing adenomas can be small and can enlarge, reimaging helped us find the possible source of aldosterone in this case. To the best of our knowledge, this is the first reported case where reimaging of the adrenal glands years after initial diagnosis of PA was helpful in identifying an adrenal nodule that was causing PA. An important factor for consideration here is the rate of growth of these types of adenomas, which to the best of our knowledge, has not been previously published. In this case, it appears that the growth rate was faster than typically would be anticipated, as it grew from not being radiographically detected to being visible 1 year later. While we are not sure why this may have occurred, it is likely that this type of presentation does happen, as most unilateral causes of PA are diagnosed by adrenal vein sampling. Therefore, in certain cases of initially CT-negative subjects with non-lateralizing AVS results, reimaging may be warranted, especially in cases where the patient is an excellent candidate for surgery and has significant morbidity from their disease.

## Consent

Written informed consent was obtained from the patient for publication of this Case report and any accompanying images. A copy of the written consent is available for review by the Series Editor of this Journal.

## Competing interests

The authors declare that they have no competing interests.

## Authors’ contributions

BTL led the acquisition of data, review of the literature and drafted the manuscript. AJH and DME reviewed the manuscript. All authors read and approved the final manuscript.
